# Magnitude, trends and causes of maternal mortality among reproductive aged women in Kersa health and demographic surveillance system, eastern Ethiopia

**DOI:** 10.1186/s12905-018-0690-1

**Published:** 2018-12-05

**Authors:** Gezahegn Tesfaye, Deborah Loxton, Catherine Chojenta, Nega Assefa, Roger Smith

**Affiliations:** 1 School of Public Health, College of Health and Medical Sciences, Haramaya University, Po Box: 235, Harar, Ethiopia; 2 Research Centre for Generational Health and Ageing, Faculty of Health and Medicine, University of Newcastle, Newcastle, Australia; 3 Mothers and Babies Research Centre, Faculty of Health and Medicine, University of Newcastle, Newcastle, Australia

**Keywords:** Maternal mortality, Reproductive aged women, Kersa HDSS, Eastern Ethiopia

## Abstract

**Background:**

Despite efforts at curbing maternal morbidity and mortality, developing countries are still burdened with high rates of maternal morbidity and mortality. Ethiopia is not an exception and has one of the world’s highest rates of maternal deaths. Reducing the huge burden of maternal mortality remains the single most serious challenge in Ethiopia. There is a paucity of information with regards to the local level magnitude and causes of maternal mortality. We assessed the magnitude, trends and causes of maternal mortality using surveillance data from the Kersa Health and Demographic Surveillance System (HDSS), in Eastern Ethiopia.

**Method:**

The analysis used surveillance data extracted from the Kersa HDSS database for the duration of 2008 to 2014. Data on maternal deaths and live births during the seven year period were used to determine the maternal mortality ratio in the study. The data were mainly extracted from a verbal autopsy database. The sample was comprised of all reproductive aged women who died during pregnancy, childbirth or 42 days after delivery. Chi-squared test for linear trend was used to examine the significance of change in rates over time.

**Results:**

Out of the total 311 deaths of reproductive aged women during the study period, 72 (23.2%) died during pregnancy or within 42 days of delivery. The overall estimated maternal mortality ratio was 324 per 100,000 live births (95% CI: 256, 384). The observed maternal mortality ratio has shown a declining trend over the seven years period though there is no statistical significance for the reduction (χ^2^ = 0.56, *P* = 0.57). The estimated pregnancy related mortality ratio was 543 per 100,000 live births (95% CI: 437, 663). Out of those who died due to pregnancy and related causes, only 26% attended at least one antenatal care service. The most common cause of maternal death was postpartum haemorrhage (46.5%) followed by hypertensive disorders of pregnancy (16.3%).

**Conclusion:**

The magnitude of maternal mortality is considerably high but has shown a decreasing trend. Community-based initiatives that aim to improve maternal health should be strengthened further to reduce the prevailing maternal mortality. Targeted information education and communication should be provided.

## Introduction

Improving women’s health and reducing maternal mortality has been a global public health priority for the United Nations international development agenda [1, 2]. Globally, the Maternal Mortality Ratio (MMR) declined from 385 in 1990 to 216 in 2015. However, during the same period, the MMR reduction in sub-Saharan Africa remained stalled and most countries in the region registered sluggish progress in reducing maternal mortality [3–5]. According to World Health Organization (WHO) estimates, even though the magnitude of MMR in Ethiopia remains high, the level has shown a steady decline from 1250 in 1990 to 353 in 2015 [3]. In the Sustainable Development Goal (SDG) period, the goal is to reduce the global MMR to less than 70 per 100,000 live births by 2030 with no country having MMR over 140 per 100,000 live births [6].

In Ethiopia, the Federal Ministry of Health (FMOH) has applied multi-pronged approaches to reducing maternal morbidity and mortality. These approaches have included improving access to and strengthening facility-based maternal health services [7]. Ethiopia’s Health Sector Development Plan target was to reduce MMR to 267 per 100,000 live births by the year 2015 but the country was unable to meet this target [8]. It was well recognized that the huge burden of maternal mortality in Ethiopia remains the single most serious challenge to the health sector [9]. In the country, the efforts to end preventable maternal mortality is at the top of the health sector’s agenda in line with the SDGs as the issue was targeted in the 2015 health sector transformation plan [10].

The highest number of maternal deaths has been reported in countries where women are least likely to deliver their babies with the assistance of skilled practitioners, such as a nurse/midwife, doctor and other health workers [11]. In many countries, it is those women who are living in rural areas, at the lowest wealth quintile and with less education who are most susceptible to maternal mortality [4, 11]. High maternal mortality levels are also an indication of deep-seated gender inequalities that hinder women’s ability to make decisions about household resources, which in turn could limit their ability to obtain social support and to access maternal health services [12].

Despite efforts at curbing maternal morbidity and mortality, developing countries are burdened with high maternal morbidity as well as mortality and are still facing the challenge of addressing the problem with very limited personnel and material resources [13]. More than 50% of all maternal deaths were from just six countries: Ethiopia, India, Nigeria, Pakistan, Afghanistan, and the Democratic Republic of Congo [14, 15]. The modelled estimate by WHO and the World Bank for Ethiopia showed a MMR of 353 per 100,000 live births in 2015 [3].

In Ethiopia, most studies on maternal mortality have been conducted in a facility setting [16–18]. Given that a small number of mothers deliver at health institutions in Ethiopia, maternal mortality measurement from facility-based data might not reflect the actual image on the ground [7, 19]. In addition, there is a paucity of information with regards to the local level magnitude and causes of maternal mortality at the community setting in Ethiopia. Furthermore, the differential results in the magnitude of maternal mortality across different estimates for Ethiopia calls for more rigorous and locally generated evidence [3, 7, 20]. This study therefore aimed to investigate the magnitude of, trends in, and causes of maternal mortality among reproductive aged women using surveillance data in a community setting in Eastern Ethiopia.

## Methods

### Study setting

The study was conducted in the Kersa HDSS site, Kersa district, Eastern Ethiopia. The HDSS is a member of INDEPTH network [21]. According to the country’s 2014 population projection, the district has an estimated total population of 205,628. The district has 38 kebeles (the smallest administrative units in Ethiopia with an average population of 5000), of which three are urban and 35 are rural kebeles [22, 23]. The Kersa HDSS baseline census was conducted in 2007 and since then it has been updated every six months, with the registration of demographic and health events. In Kersa HDSS catchment population, there are six health centres, 20 health posts, and five clinics. From 2008 to 2014, there were 12 kebeles under the Kersa HDSS and the current study considered the data that was drawn from this surveillance population.

### Study design

The study used longitudinal population based surveillance design and we carried out secondary data analysis through extracting data for the seven consecutive years (2008–2014).

### Population

All women of reproductive age at the Kersa HDSS site during the period (2008–2014) were the source population. The study population were all reproductive aged women who died during 2008–2014 and were recorded by the Kersa HDSS. Data on the deceased women who used to live in the study area for less than six months were excluded from the analysis as they were not confirmed to be permanent residents.

### Source of data and data collection methods

The primary source of the data was the Verbal Autopsy (VA) database of the Kersa HDSS. VA is a method of interviewing close relatives or caregivers of the dead person about the circumstances, signs, and symptoms that occurred before the death event and the respondent will answer in his/her own words [24]. Once the interview is completed, the VA questionnaires were passed on to at least two physicians to assign the cause of death using the International Classification of Diseases (ICD)-10 codes. After checking the agreement of physician-assigned cause of death based on VA coding, discordant cases were sent to a third physician for independent review and diagnosis. If any two of these three physicians assigned the same cause of death, then that was considered as the final cause of death; otherwise, the causes were labelled as undetermined. In addition to the information on the VA database, some basic data such as religion and ethnicity of the deceased women were obtained from the main household registration system database of the Kersa HDSS.

The data extraction procedure from the VA database was elucidated as follows. From all the deceased individuals who have VA data in the database, males of all age were excluded. Then from all the deceased women in the database, the data of all women of age less than 15 years and greater than 49 years were excluded. From all the women deaths in the reproductive age, the data of those women who were not pregnant or beyond 42 days after birth during the time of death were excluded. Finally the remaining data of all deceased women during pregnancy, birth or within 42 days of delivery were considered for the analysis.

### Data quality control

Quality assurance measures were embedded into all aspects of the surveillance process. In the Kersa HDSS, if inconsistent or missing data were detected at any step during the data collection process, the questionnaire was returned to the data collectors for checks and corrections. In addition, the supervisor selected 5% of questionnaires and visited the houses where the data were collected to check whether the information was accurate or not. The field coordinator checked 1% of the questionnaires in a similar manner. Similar measures of quality assurance procedures have also been applied to the data collection process that makes use of the VA questionnaires for a deceased person. The surveillance also made use of a standardized study tool. During the data extraction, cross-checking of the electronic version of the data with the archived hardcopy was carried out through tracing the information back on a sample of deceased women. Data cleaning and adjustments were conducted to avoid errors in the labelling or order of the variables of interest.

### Data analysis

Descriptive statistics consisting of frequency and proportion were performed to summarize the main variables. The analysis was conducted in STATA software. Some of the major statistical parameters computed were the following: MMR, pregnancy related death ratio, maternal mortality rate, lifetime risk of maternal deaths, and proportion of maternal deaths among female deaths. The overall level of MMR was calculated by dividing the total number of maternal deaths (from the VA data) from 2008 to 2014 with the total number of live births in the same period and then converted in to 100,000 live births. The level of MMR for each year starting from 2008 and up to 2014 were calculated in the same manner and the trend at different years were plotted. The temporal trend of maternal mortality was also conducted to demonstrate the seasonal variation. We used Chi-squared test to determine the significance of the trend over time. “*Pregnancy related death*” was the number of deaths of women while they were pregnant or within 42 days after termination of pregnancy irrespective of the cause divided by a total number of live births in the same period. “*Proportion of maternal deaths among female deaths*” was the number of maternal deaths divided by the total number of deaths among reproductive aged women in the same period. The “*Life Time Risk (LTR) of maternal death*” was approximated by [LTR = 1-(1-Maternal mortality rate) ^35^]. To obtain the LTR, we first calculated maternal mortality rate (Mmrate) by dividing the number of maternal deaths by the total number of reproductive aged women in the study area during the same period. The LTR of maternal death is an important measure of the cumulative loss of life due to maternal deaths over a woman’s life course [25].

## Results

### General findings

Out of the total number of reproductive aged women (34,101) during the study period, there were a total of 311 deaths. Of these, 72 (23.2%) (95% CI: 18.6, 28.2%) occurred during pregnancy or within 42 days after delivery. Out of all the women who died during pregnancy or within 42 days after delivery, 43 (59.7%) with 95% CI (47.5, 71.1%) died due to pregnancy or related causes based on the ICD codes. In the same period, the total number of live births was 13,269. Hence, based on this, the MMR was 324 per 100,000 live births (95% CI: 256, 384). The Pregnancy Related Mortality Ratio was 543 per 100,000 live births (95% CI: 437, 663), the proportion of maternal deaths among female deaths was 13.8% (95% CI: 10.2, 18.2%), and the lifetime risk of maternal death was calculated by (LTR = 1-(1-Mmrate)^35^) = 1-(1–0.00126)^35^, LTR = 1–0.95683166, LTR = 0.0432 ~ 4.3% (approximately one in 23).

### Basic socio-demographic characteristics

The mean age of the women who died due to maternal causes was 27.6 (SD = 7.5) years. The majority of the mothers who died due to maternal causes were illiterate (83.7%), married (90.7%), a house-wife (72.1%), of Muslim religion (93.1%) and Oromo by ethnicity (93.1%) (Table 1).

**Table 1:** Distribution of women who died due to pregnancy related causes by socio-demographic characteristics, Kersa HDSS, 2008–2014

Variables	All pregnancy related deaths (*n* = 72)	
	Maternal deaths, no (%)	Non-maternal deaths, no (%)
Age		
15–19	5 (11.6)	5 (17.2)
20–29	21 (48.8)	8 (27.6)
30–39	13 (30.2)	15 (51.7)
40–49	4 (9.3)	1(3.4)
Marital status		
Never married	1 (2.4)	0
Married	39 (90.7)	28 (96.6)
Widowed	3 (6.9)	1 (3.4)
Occupational status		
Farmer	5 (11.6)	4 (13.8)
House maid	4 (9.3)	2 (6.9)
House wife	31 (72.1)	20 (69)
Merchant	1 (2.3)	1 (3.4)
Student	2 (4.7)	0
Daily labourer	0	2 (6.9)
Educational status		
Illiterate	36 (83.7)	25 (86.2)
Grade 1–4	2 (4.7)	3 (10.3)
Grade 5–8	2 (4.7)	1 (3.4)
Grade 9–10	2 (4.7)	0 (0.0)
Grade 12+	1 (2.3)	0 (0.0)
Ethnicity		
Oromo	40 (93.1)	29 (100)
Amhara	3 (6.9)	0 (0.0)
Religion		
Muslim	40 (93.1)	29 (100)
Orthodox Christian	3 (6.9)	0 (0.0)
Residence		
Urban	5 (11.6)	4 (13.8)
Rural	38 (88.4)	25 (86.2)

### Magnitude and trends of maternal mortality

**Fig. 1:**
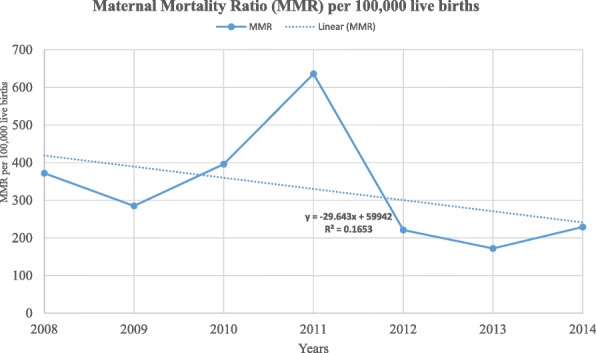
Trends of maternal mortality ratio in Kersa HDSS, 2008–2014

**Fig. 2:**
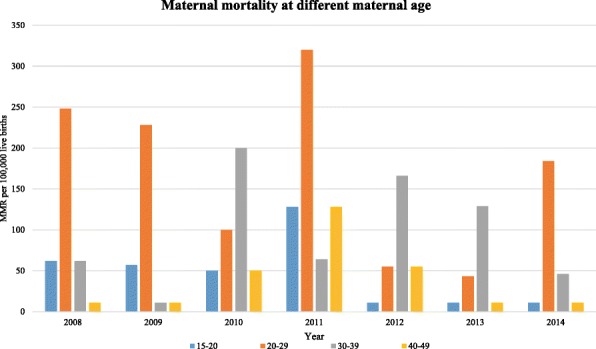
Age wise distribution of maternal mortality in Kersa HDSS, 2008–2014

**Fig. 3:**
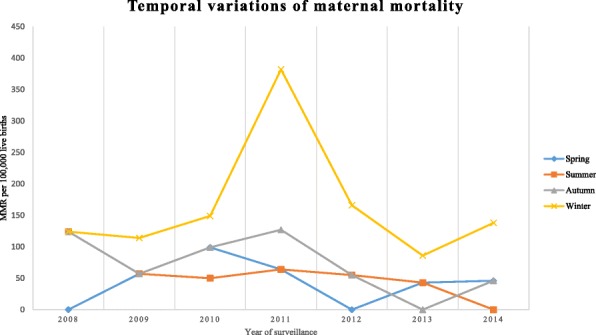
Temporal variation of maternal mortality in Kersa HDSS, 2008–2014

The overall MMR was 324 per 100,000 live births with 95% CI (256, 384) in the study area during the reference period. The number of maternal deaths per each year and the corresponding MMRs with confidence intervals are presented in Table 2. Across the study years the trend of the MMR varied, the lowest being in 2013 with 172 per 100,000 live births and the highest peak observed in 2011 with 636 per 100,000 live births. There was a slowly declining trend in MMR during the reference period in the study area with a gradient of 29.643 on linear scale (Fig. 1), though there is no statistical significance for the reduction (χ^2^ = 0.56 and *P* = 0.57). Furthermore, as shown in Fig. 2, except in 2010, 2012 and 2013, the highest rates of maternal mortality persistently occurred in the age group 20–29 years. Moreover, the study revealed an observed temporal (seasonal) variations in MMR in the study area. Throughout the study period, the highest rate of death was observed during the winter season. At the beginning of the surveillance year, the MMR overlapped at 124 per 100,000 live births for the three seasons (winter, autumn and summer). In subsequent years, however, the rate persistently became higher in the winter season until the end of the surveillance period with the highest rate observed in 2011 (Fig. 3).

**Table 2:** Annual maternal mortality ratios over the seven years period (2008 to 2014), Kersa HDSS

Year	Number of maternal deaths	Number of live births	MMR with 95% CI
2008	6	1615	372 (186, 557)
2009	5	1757	285 (114, 445)
2010	8	2019	396 (198, 502)
2011	10	1572	636 (382, 827)
2012	5	1808	221 (111, 442)
2013	4	2319	172 (60, 245)
2014	5	2179	229 (92, 335)

### Causes of maternal death

**Fig. 4:**
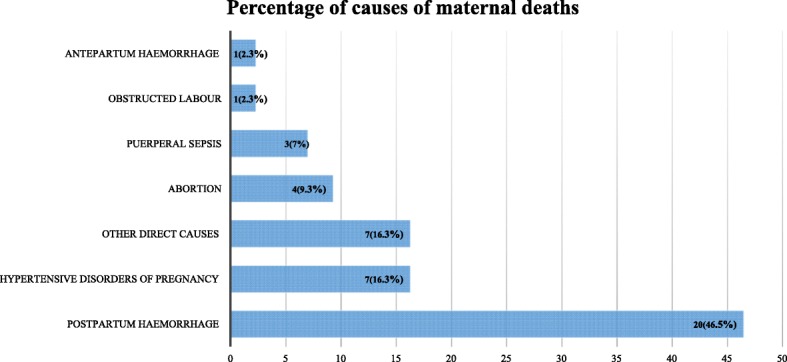
Proportion of maternal deaths by cause, Kersa HDSS, 2008–2014

**Fig. 5:**
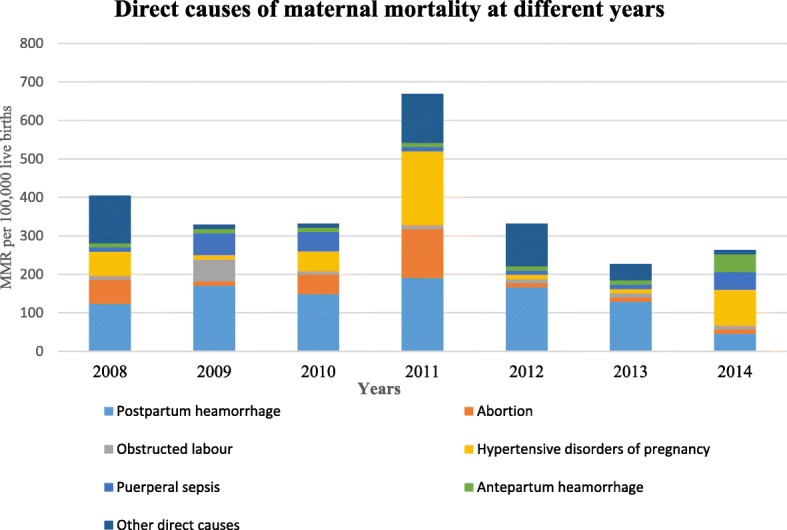
Causes of maternal mortality across various years (2008–2014), Kersa HDSS

The maternal morbidities that lead to maternal deaths were identified using the ICD codes. This was generated from the VA database using the corresponding VA code. Accordingly, the main cause of death among the 43 (59.7%) mothers who died due to pregnancy or its related causes was postpartum haemorrhage (46.5%), followed by hypertensive disorders of pregnancy (16.3%) (Fig. 4). It is worth noting that, among the pregnancy related deaths, a significant percentage (14%) of the mothers died due to partner violence and transport accidents. Though not statistically significant (*P* = 0.40), there was an observed variation in the cause of maternal death across different years (Fig. 5). For instance, though postpartum haemorrhage persisted in being the leading cause of maternal death from 2008 to 2013, in 2014 however, the leading cause of maternal death was hypertensive disorders of pregnancy.

### Place of death

Regarding the place of maternal death, most (56%) of the deaths occurred at home, followed by the hospital (33%), other places (9%), and health centre (2%). The majority (63%) of maternal deaths occurred after giving birth, and among which 18 (67%) give birth at home and 9 (21%) delivered at health facility. Among those who gave birth at home, 27% died in a hospital and other lower level health facilities.

### Previously known morbidities

Based on the information from the respondents, there were previously known morbidities among the deceased mothers such as high blood pressure (9.3%), diabetes (2.3%), malnutrition (2.3%), tuberculosis (2.3%) and other diseases such as anaemia (4.7%). With regards to injuries or accidents, only 3% of the deceased mothers were known to have a history of injuries or accidents, such as suicide and insect bites, surrounding their deaths.

### Obstetric measurements and health service use

Nearly a quarter (26%) of the deceased mothers attended at least one antenatal care consultation for their pregnancy. With regards to the timing of death during the course of pregnancy, among the deceased mothers, 27 (62.8%) died after giving birth, the majority of those (55.6%) within the first day. Among those who died after giving birth, the majority (66.7%) gave birth at home. The majority of the deliveries were assisted by untrained Traditional Birth Attendants (TBAs) (48.1%) (Table 3).

**Table 3:** Obstetric measurements of women who died due to maternal causes, Kersa HDSS, 2008–2014

Obstetric related variables (*N* = 43)	Frequency	Percent
Died during pregnancy (before delivery)	12	27.9
Gestational age at death (*n* = 12)		
First trimester	3	25
Second trimester	2	16.7
Third trimester (including in labour)	7	58.3
Died after giving birth	27	62.8
Died after undergoing abortion	4	9.3
Type of delivery (*n* = 27)		
Normal	21	67.7
Forceps/vacuum	4	19.9
Caesarean section	2	6.6
Died postnatally (*n* = 27)		
Within the first day	15	55.6
Between 1 and 7 days	8	29.6
Between 7 days −6 weeks	4	14.8
Place of delivery (n = 27)		
Home	18	66.7
Hospital	8	29.6
Health centre	1	3.7
Delivery attendants during delivery (n = 27)		
TBAs		
Untrained TBAs	13	48.1
Trained TBAs	3	11.1
Doctors	7	25.9
Nurses	2	11.1
Relatives	2	3.7

More than half (60.6%) of the deceased mothers received some treatment for the condition that led to their death. The main treatment modality the mothers received before their death was oral and injection antibiotics (46.2%). Other treatments included intravenous fluid and Oral Rehydration Salt (34.6%), nasal treatment (15.4%) such as food or fluid that passed through the nose, and one woman received blood transfusions (3.8%). With regards to the place of treatment, among the mothers who received some treatment, a substantial proportion (80.8%) received treatment at home assisted by traditional healers. However, a larger proportion (84.6%) of the mothers at same time received treatment at government clinics during the course of the health condition that led to their death. Only 2.3% of the interviewees declared that the deceased mothers had a history of smoking cigarettes.

## Discussion

Using surveillance data, this study was intended to assess maternal mortality and identify the causes of death among reproductive aged women over a seven-year period in Kersa HDSS, Eastern Ethiopia. The study showed a cumulative average MMR of 324 per 100,000 live births with a decreasing trend over the study period. The main causes of maternal death were postpartum haemorrhage (46.5%) and hypertensive disorders of pregnancy (16.3%). Most of the mothers (56%) died at home, and the majority (62.8%) of the mothers died after giving birth.

**Fig. 6:**
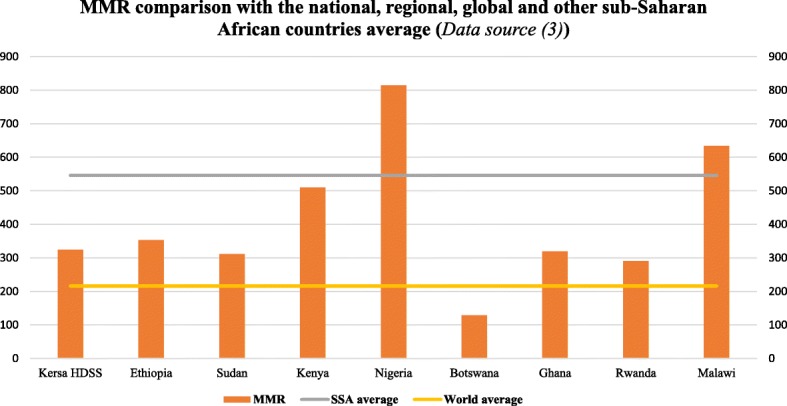
Comparison of the maternal mortality ratio with the national, regional, global and other sub-Saharan African countries average

For every 1000 live births in the study area during the seven year period, about three women died during pregnancy, childbirth or within 42 days of childbirth. This finding is almost similar to the national average (353 per 100,000 live births) but below the sub-Saharan average (546 per 100,000 live births) [3]. Even though the MMR is below the sub-Saharan Africa average, it is higher than the world average, as is the case with other sub-Saharan African countries such as Sudan, Ghana, and Rwanda (Fig. 6) [3]. The overall cumulative average of maternal mortality in the present study is considerably high and is deemed to require government action. However, the observed MMR across the seven-year period appears to decline. The result of the present study is almost in agreement with the finding that was reported from a review of studies conducted in Ethiopia, where the level of maternal mortality was found to decline slightly [17]. The decreasing level of maternal mortality in this study might be related to the current government efforts to improve maternal health by implementing community-based programs that involve community mobilization. Moreover, it might also be explained by the fact that the population under surveillance (through the HDSS) most likely have better awareness and use of maternal health care, which could potentially lead to a lowered MMR. Furthermore, it was found that residing in a HDSS site has a positive influence on maternal health [26].

Nonetheless, the trend of maternal mortality has varied slightly over the years. For instance, there was an increase in MMR in 2011 but a decrease in 2013. The reasons for this are not clear but may have something to do with the relatively higher number of pregnancies and increased number of poor pregnancy outcomes in 2011 compared to 2013. There were more pregnancies in that year, which might be associated with a high agricultural production in the preceding year that enhanced an increased number of families formed among young couples in the district. Moreover, there was a high number of pregnancy failures and still-births in the same year [21]. In connection with this, in the same year compared to other years, the absolute number of maternal deaths may have increased. In addition, the study findings revealed that there had been temporally heterogeneous pattern in rates of maternal death, where MMR was highest during the winter season throughout the study period. The finding highlights that since the winter season has heavy rain falls, the road may get muddy, the rivers might become full and rural bridges could be damaged. Hence the parturient family might not be able to transport the women to a health facility for delivery care and thus most women remain at home to deliver their baby, which might have contributed to the higher rates of maternal death. Moreover, during the rainy season there is an increased rate of malaria transmission, and pregnant women could in turn develop adverse maternal disorders (anaemia and eclampsia) which subsequently contribute to high rates of maternal deaths. It has been shown that malaria-associated anaemia and eclampsia tend to increase during the rainy season among pregnant women in sub-Saharan Africa [27].

Generally, we found the leading cause of maternal death during the study period to be postpartum haemorrhage. This finding is in accordance with other research results from developing countries and sub-Saharan Africa where haemorrhage is the leading direct cause of maternal death [28, 29]. A similar finding was also reported from a community-based study in northern Ethiopia [30]. The finding of the current study, however, is in contrast with a systematic review of studies in Ethiopia which showed the major cause of maternal death to be obstructed labour (36%) followed by haemorrhage (22%) [16]. This difference may be partly explained by the fact that the review only included facility-based studies where most pregnant women came to a health facility very late, with advanced complications such as obstructed labour which leads to prolonged labour [16, 31]. Another possible explanation for this may be that, as the VA data used in the present study is prone to misclassification of maternal deaths, the examiners might have misdiagnosed obstructed labour that leads to haemorrhage. The mothers, however, might have died due to initially developing obstructed labour which is typically not the cause of death but rather due to the haemorrhage that resulted from prolonged labour.

In the current study, there were 543 pregnancy-related deaths per 100,000 live births during the seven-year period. Though the majority of the pregnancy-related deaths were due to causes related to pregnancy or childbirth, non-obstetric causes such as partner violence and transport accidents were also contributors. This highlights that partner violence, including murder and other injuries or accidents to women, contributes to the rates of death among pregnant and postpartum women to a considerable extent in Ethiopia. This underscores the need to design programs that could address issues of gender-based violence at the community level to synergize with current efforts to improve maternal health. The results of the study are similar to a study conducted in Mozambique, in which it was reported that a combination of partner violence and injury were the fourth leading cause of maternal death [32]. The finding further highlights that the social status of women in the community might be a root factor for the high rate of maternal mortality in Ethiopia.

The findings of this study demonstrated that the lifetime risk of maternal death is nearly 1 in 23. This result is lower than a study finding from a community-based survey in southern Ethiopia which was conducted using the sisterhood method, where women have a one-in-ten lifetime risk of deaths [33]. However, this finding is higher as compared to the sub-Saharan African (1 in 39) and national level (1 in 52) [34, 35]. The reason for the observed high LTR of maternal death in the present study might be related to the less precise estimation of the indicator using Mmrate.

In this study, the majority (62.8%) of the mothers died after giving birth and more than half (55.6%) died within the first day. These findings indicate that the first few hours and days after giving birth are a critical period during which mothers should receive immediate attention from health care providers at a health facility to avert catastrophic maternal deaths. The aggregation of maternal death around delivery or immediately after delivery also means that mothers should have access to health facilities to receive skilled care during this period.

In the present study, more than half (60.5%) of the deceased mothers had sought health care services for the health condition that led to their death. However, a substantial proportion (80.8%) of them sought the service from traditional healers at home. This has implications for maternal health behavioural change programs. Traditional healers, including TBAs, still play a paramount role in rendering services at the community level but it has been shown that they are not effective in improving maternal health even when trained [36, 37]. Yet the reliance of local women on TBAs emphasizes the need to understand, at the grassroots level, why uptake of skilled delivery care is low in comparison with TBA utilisation. Perhaps strategies should be designed to provide focused training to TBAs so as to make them capable of recognizing the critical time to seek health care for women in their village [38]. Therefore, there is a need to consider revisiting the strategies for training of TBAs to make them contribute towards the improvement of maternal health.

### Implications of the study

The measurement of maternal mortality using data from the direct surveillance system is the current gold standard method of determining the MMR. Using direct surveillance methods in the current study, it was possible to estimate the MMR. Hence, the study demonstrates the utility of estimating maternal mortality based on HDSS data to inform policy and enable locally appropriate program development. Despite efforts to maintain the data quality at every step of the surveillance process at Kersa HDSS, there was still some misclassification of deaths, which could be partly attributed to misreporting. For the data to be strong enough to support evidence-based decision making, it is crucial that data collection systems in HDSS sites institute ways to improve reporting from the community.

### Limitations of the study

As the study used secondary data, there were incomplete or mislabelled variables, restricted variable data, inconsistent values, and missing records. In addition, the VA codes used to assign the cause of death were mainly limited to the direct causes, rather than the indirect causes. This could be related to the fact that the cause of death was determined by physicians using the VA questionnaire, which depends on the subjective response of the interviewee. This might most likely suffer from respondents’ information bias, which may lead to misclassification of the underlying cause of death. Due to the sensitive nature of the issue, abortion-related maternal deaths were likely to be underreported. Using the current data, we are unable to make inferences that compare the women who died with the women who survived childbirth. Moreover, the use of a small sample for the analysis made it difficult to draw inferences to the general population. However, our intent was to describe maternal mortality at the local level in the Kersa HDSS.

## Conclusion

The magnitude of maternal mortality is considerably high, though it has shown a declining trend. The major causes of maternal mortality were postpartum haemorrhage and hypertensive disorders of pregnancy. Community-based initiatives should be strengthened to further reduce the prevailing maternal mortality. Targeted information education and communication should be provided to illiterate housewife women in their twenties. The health messages targeting these group of mothers should be tailored to their needs and match their level of literacy in order to bring better health outcomes. Future interventions on maternal health in this setting should also be tailored in such a way that women are educated through existing mother peer groups or Women’s Development Army networks at the village level. Moreover, strategic actions are required to promote skilled delivery care attendance and attention should be given to availing community based trained delivery assistants in rural communities.

## Abbreviations

CI: Confidence Interval
EPHA: Ethiopian Public Health Association
FMOH: Federal Ministry of Health
HDSS: Health and Demographic Surveillance System
ICD: International Classification of Diseases
LTR: Life Time Risk
MMR: Maternal Mortality Ratio
Mmrate: Maternal mortality rate
SDG: Sustainable Development Goal
TBA: Traditional Birth Attendant
VA: Verbal Autopsy
WHO: World Health Organization
